# An integrative single-cell atlas for exploring the cellular and temporal specificity of genes related to neurological disorders during human brain development

**DOI:** 10.1038/s12276-024-01328-6

**Published:** 2024-10-03

**Authors:** Seoyeon Kim, Jihae Lee, In Gyeong Koh, Jungeun Ji, Hyun Jung Kim, Eunha Kim, Jihwan Park, Jong-Eun Park, Joon-Yong An

**Affiliations:** 1 Department of Integrated Biomedical and Life Science, Korea University, Seoul, Republic of Korea; 2 L-HOPE Program for Community-Based Total Learning Health Systems, Korea University, Seoul, Republic of Korea; 3 School of Biosystem and Biomedical Science, College of Health Science, Korea University, Seoul, Republic of Korea; 4 Department of Biomedical Sciences, College of Medicine, Korea University, Seoul, Republic of Korea; 5 Department of Anatomy, College of Medicine, Korea University, Seoul, Republic of Korea; 6 Department of Neuroscience, College of Medicine, Korea University, Seoul, Republic of Korea; 7 BK21 Graduate Program, Department of Biomedical Sciences, College of Medicine, Korea University, Seoul, Republic of Korea; 8 School of Life Sciences, Gwangju Institute of Science and Technology (GIST), Gwangju, Republic of Korea; 9 Graduate School of Medical Science and Engineering, Korea Advanced Institute of Science and Technology (KAIST), Daejeon, Republic of Korea

**Keywords:** Neuroscience, Computational biology and bioinformatics

## Abstract

Single-cell technologies have enhanced comprehensive knowledge regarding the human brain by facilitating an extensive transcriptomic census across diverse brain regions. Nevertheless, understanding the cellular and temporal specificity of neurological disorders remains ambiguous due to developmental variations. To address this gap, we illustrated the dynamics of disorder risk gene expression under development by integrating multiple single-cell RNA sequencing datasets. We constructed a comprehensive single-cell atlas of the developing human brain, encompassing 393,060 single cells across diverse developmental stages. Temporal analysis revealed the distinct expression patterns of disorder risk genes, including those associated with autism, highlighting their temporal regulation in different neuronal and glial lineages. We identified distinct neuronal lineages that diverged across developmental stages, each exhibiting temporal-specific expression patterns of disorder-related genes. Lineages of nonneuronal cells determined by molecular profiles also showed temporal-specific expression, indicating a link between cellular maturation and the risk of disorder. Furthermore, we explored the regulatory mechanisms involved in early brain development, revealing enriched patterns of fetal cell types associated with neuronal disorders indicative of the prenatal stage’s influence on disease determination. Our findings facilitate unbiased comparisons of cell type‒disorder associations and provide insight into dynamic alterations in risk genes during development, paving the way for a deeper understanding of neurological disorders.

## Mapping temporal specificity of disorder risk genes in brain

The growth of the human brain is a complicated process that begins before birth and continues into young adulthood. Researchers focused on how genes related to brain disorders are expressed in different cells over time. They gathered data from 114 human brain samples, creating a single-cell atlas that traces brain growth from early fetal stages to adulthood. The results showed distinct patterns of gene expression linked to disorders like autism and developmental delay, especially in neurons during early growth. The study also emphasized the role of glial cells in brain conditions, such as Alzheimer’s and Parkinson’s disease, by showing specific gene expression patterns in these cells related to the disorders. Researchers conclude that their single-cell atlas greatly improves our understanding of brain growth and the molecular mechanisms behind brain disorders.

This summary was initially drafted using artificial intelligence, then revised and fact-checked by the author.

## Introduction

The human nervous system undergoes a gradual and complex developmental process that spans several decades, beginning with embryogenesis and continuing through infancy, childhood, adolescence, and young adulthood. This prolonged developmental period involves the formation of a myriad of functionally distinct cell types, circuits, and regions. Recent advances in single-cell technology have surpassed conventional regional analyses to include comprehensive surveys of the entire human brain. Large-scale studies such as the Brain Initiative Cell Census Network have substantially aided in constructing detailed brain maps using single-nucleus RNA-sequencing analysis^1–4^. Despite these efforts, the extant literature has several limitations, including the lack of donor-specific diversity in terms of brain development and the absence of temporal dimensions in existing brain atlas studies. These gaps underscore the necessity for enhanced, comprehensive approaches to delineate the intricate developmental processes in the human brain.

The examination of cell types can elucidate the relevant pathology of neurological disorders, which is highly clinically important. Because risk genes or genetic variants associated with neurological disorders are heterogeneous, understanding the cell types associated with converging their risk would increase our understanding of the complex etiology and pathophysiological mechanisms underlying the disorder. De novo variants in autism disrupt neuronal genes that are overexpressed in the prefrontal cortex during the mid-fetal period^5^, indicating that risk perturbation at the molecular level precedes its clinical onset^6^. Furthermore, single-cell transcriptomics has the potential to suggest a novel molecular subtype that is not apparent through traditionally observed symptoms. A large-scale single-cell study of Alzheimer’s disease postmortem brains was able to identify a novel excitatory neuron cell type enriched in the cohesin complex and DNA damage response factors and its association with cognitive impairment in patients^7^ and its genetic associations with noncoding mutations^8^. This single-cell transcriptome approach elucidates the fundamental characteristics of these disorders and facilitates the development of more effective and personalized treatments. These findings underscore the imperative for comprehensive single-cell transcriptome studies to dissect the roles of neurological genes across diverse cell types, further enhancing our understanding of brain disorders. However, existing public datasets often include a limited number of samples and donors, underscoring the necessity of creating an extensive resource to better understand and address neurological disorders comprehensively.

This study aimed to present the brain transcriptome at the single-cell level (BTS), a single-cell atlas for developing human brains, and evaluate the cellular and temporal specificities of neurological disorder genes. Data from eight previously published single-cell transcriptomics studies were integrated to create a dataset comprising 114 human postmortem brain samples from 80 donors, spanning from the early fetal stages (7 gestational weeks) to late adulthood (90 years old). Our atlas offers a comprehensive resource for elucidating the developmental trajectory of the human brain and identifying various cell types that represent distinct temporal windows of development. Furthermore, leveraging this dataset, we conducted an in-depth exploration of cell type-specific genes implicated in neurological disorders and delineated the molecular mechanisms underlying neurodevelopmental disorders and age-related neurological conditions. This study advances human brain research and provides valuable insights into normal and pathological neurological processes throughout the human lifespan.

## Materials and methods

### Collection of single-cell transcriptome datasets

Single-cell and single-nucleus RNA-sequencing datasets were obtained in raw count matrices from publicly available sources. We collated 8 datasets to ensure a complete representation of brain development, spanning both the prenatal and postnatal stages. Neurotypical samples were selected from each dataset to reflect normal brain development. Our datasets included 114 samples from 80 donors obtained from the Allen Brain Map Cell Types Database, the European Genome-phenome Archive (EGA), the Gene Expression Omnibus (GEO), and the Human Cell Atlas Data Portal (Supplementary Table 1a). Count matrices were collated from Allen Brian Human_M1 10X data (https://portal.brain-map.org/atlases-and-data/rnaseq/human-m1-10x, *n* = 2), Braun et al.^9^ (EGAD00001006049, *n* = 19), Cameron et al.^10^ (EGAS00001006537, frontal cortex, and ganglionic eminence region, *n* = 3), Hardwick et al.^11^ (GSE178175, *n* = 2), Herring et al.^12^ (GSE168408, *n* = 24), Morabito et al.^13^ (GSE174367, *n* = 7), Nagy et al.^14^ (GSE144136, *n* = 17), and Zhu et al.^15^ (GSE202210, *n* = 6). For the Herring et al. dataset, cells annotated as poor-quality clusters in the original publication were excluded. In the Morabito et al. dataset, cells lacking sample information were excluded. For the Braun et al. dataset, sex information was inferred based on XIST expression. Metadata for each sample was collected and harmonized in the format of the Human Cell Atlas, including the source dataset, donor ID, sample ID, sequencing platform, library batch, sex, age, stage, race, hemisphere, brain region, postmortem interval (PMI), and diagnosis (Supplementary Table 1b). Age information was formatted in days, with the fetus’s age described in terms of gestational age, assuming a 40-week pregnancy from the last menstrual period. For datasets recording fetal age in postconceptual age (assuming a 38-week pregnancy), 14 days were added to convert to gestational age. The samples were categorized into 11 developmental stages based on the definitions by Kang et al.^16^.

### Quality control and integration

The raw count matrices from distinct datasets were concatenated into a single dataset. Gene names were standardized using a gene symbol dictionary derived from NCBI and HGNC, excluding genes without valid annotations. Cells with small numbers of genes (<50 genes) and large proportions of mitochondrial genes (>30% of the total gene count) were filtered out. Doublet detection was performed using Scrublet^17^ (v0.2.1), and putative doublets were removed. To ensure sample balance across datasets, 50 samples from the Braun et al. dataset were randomly selected for analysis. Normalization and log transformation were conducted using Scanpy^18^ (v1.8.2). Highly variable genes were selected within each sample and merged to prevent batch-specific biases. This process involved calculating highly variable genes within each sample, sorting them by the number of samples in which they were identified, and selecting the top 5000 genes found in most samples. Datasets were merged across different samples using scvi-tools^19^ (v1.0.3), and the batch effect was adjusted by setting the batch key as the sample ID and additional categorical covariate keys, including the source dataset, assay information (single-cell or single-nuclei), and library kit (10 × 3’ v2 or 10 × 3’ v3). The latent representation for each cell was used to compute the nearest neighbor distance matrix and construct a neighborhood graph. The Leiden algorithm was used for clustering with a resolution of 0.6.

### Annotation of clusters

The major cell types were identified based on annotations from original studies and expression profiles of cell type marker genes defined by the Allen Brain Institute^20^: SLC17A7 for excitatory neurons; GAD1 for inhibitory neurons; FGFR3 for astrocytes; TYROBP for microglia; OPALIN for oligodendrocytes; PDGFRA for oligodendrocyte progenitor cells (OPCs); NOSTRIN for endothelial cells; HES1 and SOX2 for radial glia; and NHLH1 and NEUROD6 for neuroblasts. Cell subtypes for neurons were defined by layer markers for excitatory neurons (LINC00507 for layers 2–3; RORB for layers 3–5; FEZF2; and THEMIS for layers 4–6) and branch markers for inhibitory neurons (PVALB and SST for the medial ganglionic eminence (MGE); LAMP5, VIP and ADARB2 for the caudal ganglionic eminence (CGE)). Each cluster was further annotated by determining the most cluster-specific marker, exhibiting the greatest fold change with a false discovery rate (FDR) < 0.05 compared with all other cells detected in at least 25% of the cells within the cluster (Supplementary Table 2). The Wilcoxon rank-sum test was used for differential testing.

### Gene set enrichment test for neurological disorders and glioblastoma

A comprehensive set of risk genes associated with various neurological disorders and conditions identified in large-scale exome studies or genome-wide association studies (GWASs) was systematically collated. The risk genes were subjected to enrichment tests with cluster-specific DEGs with a threshold of FDR < 0.05 and a log_2_-fold change >0.2. A one-sided Fisher’s exact test with multiple comparisons was applied. A detailed description of the method is included in the Supplementary Methods.

### Pseudotime and trajectory analysis

Pseudotime analysis was performed using Palantir^21^. The subset of each cell type of interest was reprocessed before analysis. Diffusion maps were derived from the embeddings. Pseudotime computation and trajectory construction were conducted. Gene trends were subsequently computed for each lineage, and gene expression was illustrated across pseudotime for temporal investigation. A detailed description of the method is included in the Supplementary Methods.

### Analysis of gene expression profiles across developmental ages

Pseudobulk aggregation of the expression matrix was performed using decoupler^22^ (v1.5.0). The expression profiles were summarized across cells per sample ID and Leiden cluster by calculating the mean log-normalized count. To ensure data quality, samples harboring a minimum of 10 cells and 1000 accumulated counts were exclusively considered for pseudobulk aggregation. Trend lines for samplewise expression were generated by fitting using the Loess function with a span of 0.4, enabling a smooth representation of expression trends across developmental ages. Leiden clusters containing more than 4600 cells were included to capture robust population dynamics, and cells from the Nagy et al. dataset were excluded because the exact age of the samples was unknown.

### Inference of gene regulatory networks and enriched signaling pathways

Inference of gene regulatory networks involved aggregating 393,060 cells into 5000 meta-cells using SEACells (v0.3.3). Transcription factor regulatory networks were inferred using pySCENIC (v0.12.1), with regulon activities calculated and visualized as heatmaps for each SEACell. Gene sets for hormonal regulation, kinase-mediated pathways, and immune signaling were analyzed from the Reactome database, computing module scores by averaging expression levels. A detailed description of the method is included in the Supplementary Methods.

## Results

### Generation of a single-cell atlas for developing human brains

**Fig. 1:**
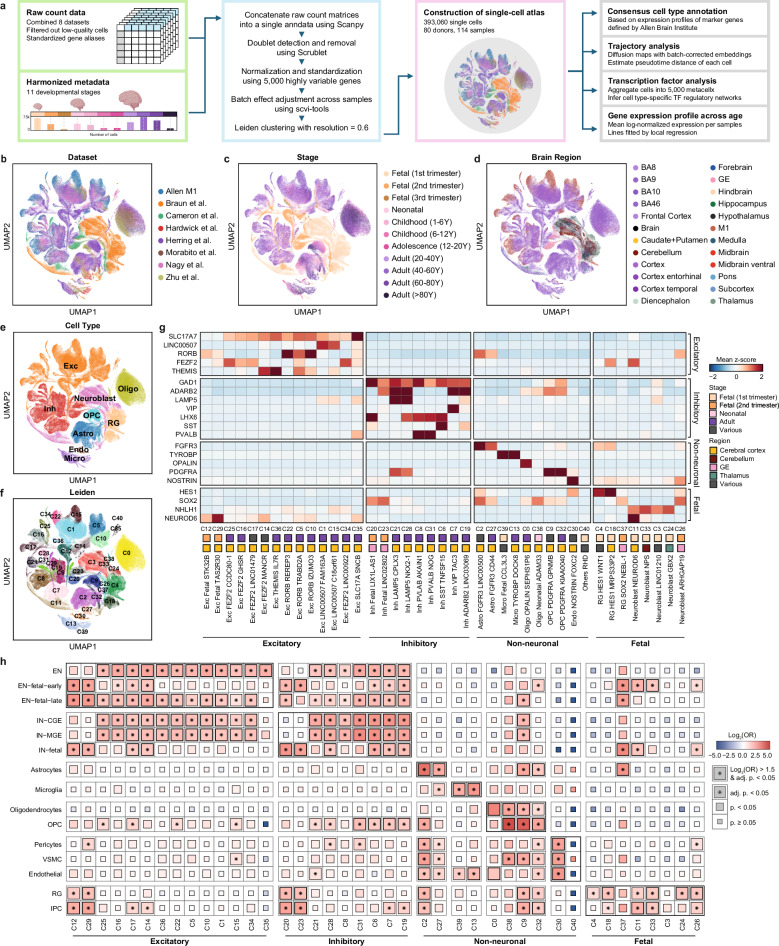
Integrated single-cell atlas of the developing human brain. **a** Schematic of atlas construction and downstream analysis. Uniform manifold approximation and projection (UMAP) of the atlas, colored by (**b**) integrated datasets, (**c**) developmental stages, (**d**) brain regions described in original studies, (**e**) major cell types, and (**f**) Leiden clusters. **g** Taxonomy of 41 Leiden clusters based on the scaled expression of marker genes. The stages and regions with the highest proportions of cells (>35%) were designated. **h** Gene set enrichment test for the established cell type marker genes sourced from a previous single-cell transcriptome study^23^. A one-sided Fisher’s exact test was used to compute statistics with multiple comparisons by Bonferroni correction.

We established a single-cell atlas for developing human brains by compiling single-cell RNA sequencing (scRNA) and single-nucleus RNA sequencing (snRNA) datasets from eight studies comprising 80 donors and 114 postmortem brain samples (Fig. 1a, b, Supplementary Fig. 1). This comprehensive single-cell atlas integrates expert-curated, quality-assured, and preanalyzed datasets from publicly available studies on the pre- and postnatal periods of the human brain. We selected samples that had not been previously reported for neurological disorders, assuming neurotypical brains. The samples encompassed a wide range of developmental stages from 7 gestational weeks to 90 years of age and diverse brain regions (Fig. 1c, d). As the datasets were heterogeneous in terms of data quality, gene name representation, and sample metadata, we rigorously conducted a quality control process for the datasets and created a consensus notation for the sample metadata (Supplementary Table 1). To mitigate the batch effect when combining these datasets, we matched gene names across the datasets and integrated the raw matrix files of the single-cell datasets using the scVI tool^19^ (Supplementary Fig. 2). Overall, the single-cell atlas integrated 393,060 single cells and 41 clusters, which were annotated as 10 major cell types and 22 cell subtypes, based on the Allen Brain Institute classifications^20^ (Fig. 1e–g).

With respect to excitatory neurons, we identified cell subtypes based on developmental stages and cortical layers. We characterized 11 clusters of cortical layer-specific excitatory neurons expressing markers for cortical layers 2–3 (LINC00507), layers 3–5 (RORB), and layers 4–6 (FEZF2 and THEMIS). Consequently, there were two clusters (C1 and C15) for layers 2–3, three clusters (C5, C10, and C22) for layers 3–5, and six clusters (C14, C16, C17, C25, C34, and C36) for layers 4–6. Additionally, we identified two clusters that were predominantly present in the fetal second trimester (C12 and C29). C12 displayed a dynamic composition spanning from 14 postconception weeks to 35 months after birth, which could offer insights into the fetal-to-neonatal transition of excitatory neurons. Inhibitory neurons were classified into cell subtypes based on marker expression of the caudal ganglionic eminence (CGE) branch (ADARB2, LAMP5, and VIP) and medial ganglionic eminence (MGE) branch (LHX6, PVALB, and SST). We characterized seven clusters of branch-specific inhibitory neurons. In the CGE branch, two clusters (C21 and C28) expressed LAMP5, one cluster (C7) expressed VIP, and one cluster (C19) expressed ADARB2. In the MGE branch, two clusters (C8 and C31) expressed PVALB, and one cluster (C6) expressed SST. Furthermore, we identified two clusters enriched in fetal inhibitory neurons (C20 and C23) that were prevalent in the second trimester. These clusters presented distinct expression patterns of branch markers. C20 was distinguished by the expression of LHX6, whereas C23 exhibited the expression of ADARB2, indicating their unique developmental origins.

Our single-cell atlas delineates clusters specific to various developmental stages. Clusters of radial glia (C4, C18, and C37) and neuroblasts (C3, C11, C24, C26, and C33) were predominantly present during the fetal first and second trimesters. Microglia and oligodendrocytes were subdivided into two clusters, each of which was based on developmental stage. We found that one microglial cluster was prevalent in the adult stage (C13), and the other was predominant in the fetal first trimester (C39). For oligodendrocytes, one cluster prevailed in the adult stage (C0), whereas the other predominated in the neonatal stage (C38). Astrocytes and OPCs were subdivided into two clusters. One cluster (C2 for astrocytes and C9 for OPCs) exhibited a mix of developmental stages, whereas the other cluster (C27 for astrocytes and C32 for OPCs) predominantly represented adult-stage cells.

We further validated our cluster annotation by evaluating the enrichment of cluster-specific differentially expressed genes (DEGs) with cell type-specific DEGs identified in the latest single-cell study of the developing human brain^23^ (Fig. 1h, Supplementary Table 2). We observed significant overlaps between fetal excitatory neurons (C12, C29) and previously identified early fetal excitatory neurons (EN-fetal-early) (C12, odds ratio [OR] = 7.81, adjusted *p* value = 9.98 × 10^−21^; C29, OR = 6.35, adjusted *p* value = 3.08 × 10^−20^), as well as late fetal excitatory neurons (EN-fetal-late) (C12, OR = 8.15, adjusted *p* value = 1.51 × 10^−29^; C29, OR = 7.55, adjusted *p* value = 9.57 × 10^−36^). We found a significant overlap between fetal inhibitory neurons (C20, C23) and previously identified fetal inhibitory neurons (IN-fetal) (C20, OR = 8.38, adjusted *p* value = 1.25 × 10^−9^; C23, OR = 6.70, adjusted *p* value = 8.52 × 10^−6^). Radial glia, neuroblasts, and nonneuronal cell types also exhibited significant enrichment with cell type-specific DEGs, further confirming our classification results. Regarding C38, which predominantly consists of oligodendrocytes present during the neonatal stage, significant overlap was observed with both oligodendrocytes (OR = 6.25, adjusted p value = 1.25 × 10^−2^) and OPCs (OR = 28.8, adjusted *p* value = 3.15 × 10^−33^), suggesting an ongoing differentiation process toward mature oligodendrocytes within this cluster. Overall, these results validate the annotation and underscore the robustness and fidelity of our atlas in capturing the dynamic landscape of human brain development.

### Cellular landscape of neurodevelopmental disorder genes in early neuronal lineages

**Fig. 2:**
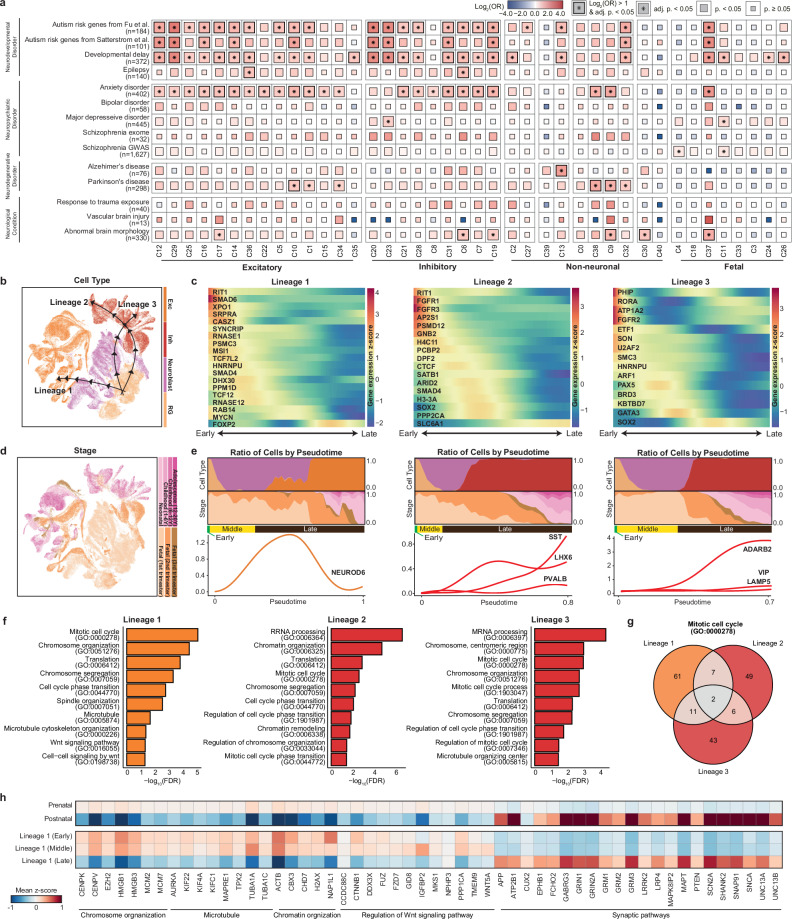
Cellular trajectories of neurodevelopmental disorder risk genes in neuronal lineages. **a** Gene set enrichment test for neurological disorder genes. A one-sided Fisher’s exact test was used to compute statistics with multiple comparisons by Bonferroni correction. **b** UMAP visualizations of estimated developmental lineages in neuronal cell types. **c** Expression profiles of neurodevelopmental disorder risk genes across pseudotime for each lineage. **d** UMAP visualizations of developmental stages in neuronal cell types. **e** Distribution of cells by major cell type and developmental stage across pseudotime and temporal patterns of late neuronal IPC markers (NEUROD6), MGE-derived inhibitory neuron markers (SST, LHX6, and PVALB), and CGE-derived inhibitory neuron markers (ADARB2, VIP, and LAMP5). The cells in the first one-third portion of the cells with pseudotime close to 0 are labeled “Early”, the subsequent portion is labeled “Middle”, and the final one-third of the cells with the latest pseudotime are designated “Late”. **f** Functional annotations for lineages with significantly enriched biological processes with multiple comparisons by FDR. **g** Venn diagram illustrating the number of overlapping genes involved in the mitotic cell cycle pathway across lineages. **h** Heatmap depicting the z score-normalized expression of genes involved in lineage timepoint-specific biological pathways. Synaptic pathways include genes involved in chemical synaptic transmission (GO:0007268) and the synaptic membrane (GO:0097060).

Over the past decade, large-scale genomic studies have identified risk genes associated with neurological disorders and implicated substantial locus heterogeneity in the underlying etiology. Unraveling the intricate temporal patterns of risk genes is crucial for deciphering the underlying pathological mechanisms and identifying the most relevant cell types and developmental stages implicated in disease pathogenesis. To investigate the cellular and temporal specificity, we compared the risk genes associated with these disorders with genes enriched in specific cell subtypes (Fig. 2a, Supplementary Table 3a). We prioritized 14 sets of genes previously reported to be associated with neurological disorders in large-scale genomic studies and examined their expression profiles in our single-cell atlas. Consequently, distinct patterns of gene enrichment were detected in a cell type-specific manner. Risk genes for neurodevelopmental disorders, including autism, developmental delay, and epilepsy, are predominantly expressed in neuronal cell types. The autism and developmental delay risk genes were considerably enriched in both excitatory and inhibitory neuronal cell types, which is consistent with previous findings^24–26^ (Supplementary Table 3b). Genes associated with epilepsy were specifically enriched in excitatory neurons located in cortical layers 4–6 (C36) and SST-expressing inhibitory neurons (C6).

We further investigated the cellular lineages underlying the neuronal clusters and the lineage-specific patterns of neurodevelopmental disorder risk genes. The pseudotime trajectory built on cellular identity reflected the clustering patterns of cells differentiating from radial glia to neuroblasts, eventually committing to mature neurons under the age of 20 years (Supplementary Fig. 3). Three distinct lineages diverged from radial glia and differentiated into excitatory and inhibitory neurons (Fig. 2b), each enriched for specific risk genes (Fig. 2c) and encompassing cells across various developmental stages (Fig. 2d). These lineages were characterized as those destined for excitatory neurons (lineage 1), MGE-derived inhibitory neurons (lineage 2), and CGE-derived inhibitory neurons (lineage 3) (Fig. 2e).

Lineage 1 represents a differentiation trajectory from radial glia to excitatory neurons, marked by the transient expression of NEUROD6 as neuroblasts transition into excitatory neurons. Among the autism risk genes, those involved in transcriptional regulation, such as RNASE1 and TCF7L2, are predominantly expressed in the early stages of this lineage. Most autism- and developmental delay-related genes peaked during the initial phase of the lineage. In contrast, RAB14 and MYCN, other risk genes for developmental delay, exhibited peaks in later stages, which coincided with neuroblast differentiation into excitatory neurons during the fetal second trimester. FOXP2, a risk gene for both autism and developmental delay, was highly expressed at the end of the lineage, particularly in the postnatal stage. The Wnt signaling pathway (GO:0016055) mediated by TCF7L2 was significantly enriched in this lineage (FDR = 4.79 × 10^−2^) (Fig. 2f, Supplementary Table 4a), suggesting its involvement in the pathophysiology of autism and developmental delay. Lineage 1 is also characterized by enrichment in the cell cycle and translation processes, implying increased cellular activity and proliferation, which are essential for neurogenesis and neuronal differentiation. Although the enrichment of genes related to the mitotic cell cycle (GO:0000278) was recurrent in other lineages, the low number of overlapping genes confirmed that the lineage-specific characteristics were distinct from those of other lineages (Fig. 2g).

To further elucidate the distinctions among the pseudotime stages within this differentiation lineage, we compared cells at earlier, middle, and later pseudotime points along with their enriched biological pathways (Fig. 2h, Supplementary Table 4d–f). Cells transitioning from early to middle pseudotime stages were enriched predominantly in pathways related to cellular infrastructure, such as chromosome organization (early, FDR = 1.74 × 10^−5^; middle, FDR = 2.26 × 10^−8^) and microtubule-based processes (early, FDR = 7.17 × 10^−3^; middle, FDR = 3.61 × 10^−2^). Genes involved in these pathways, including centromere proteins (CENPK and CENPV), high mobility group boxes (HMGB1 and HMGB3), and tubulin alpha (TUBA1A and TUBA1C), were more highly expressed in early- and middle-stage cells than in later-stage cells. Nonetheless, cells in the middle pseudotime stage were also specifically enriched in the regulation of the Wnt signaling pathway (FDR = 4.15 × 10^−2^), which was mediated by the upregulation of genes such as FZD7, GID8, IGFBP2, TMEM9, and WNT5A. Cells in the later pseudotime stage exhibited specific enrichment in synaptic pathways, characterized by the upregulation of several neuronal markers, including CUX2, glutamate metabotropic receptor genes (GRM1, GRM2, and GRM3), and several autism risk genes, including PTEN, SCN2A, and SHANK2. These results indicate that essential biological processes are dynamically regulated throughout neurodevelopment, highlighting the temporal specificity of differentiation.

Lineages 2 and 3 represented the differentiation trajectory from radial glia to inhibitory neurons, diverging into MGE-derived (C20) and CGE-derived (C23) neurons (Supplementary Fig. 3). This divergence is distinguished by the increased expression of the MGE-branch markers SST, LHX6, and PVALB in lineage 2 and the increased expression of the CGE-branch markers ADARB2, VIP, and LAMP5 in lineage 3. In lineage 2, regarding autism risk genes involved in neuronal communication, AP2S1 exhibited initial expression, whereas SLC6A1 was highly expressed in a later lineage. Risk genes for developmental delay, including CTCF, SATB1, ARID2, SMAD4, H3-3A, SOX2, and PPP2CA, were coherently expressed during the transition phase from neuroblasts to fetal inhibitory neurons around the fetal second trimester, suggesting a heightened risk of disorder at this stage of neural development. In lineage 3, risk genes associated with developmental delay, including PHIP, RORA, ATP1A2, FGFR2, ETF1, SON, U2AF2, SMC3, and HNRNPU, coherently peaked during the initial phase of the lineage. PAX5, which represents an autism risk gene involved in gene expression regulation, peaked during the middle phase of this lineage within neuroblast cells of the fetal first trimester. Moreover, other risk genes for developmental delay, such as BRD3, KBTBD7, and GATA3, also peaked during this stage, whereas SOX2 displayed high expression in a later lineage. Like lineage 1, lineages 2 and 3 presented significant enrichment in pathways associated with chromosomal organization and mitotic cell cycle processes (Supplementary Table 4b, c). Nonetheless, few genes overlapped between the common pathways, implying that lineage-specific genes distinctly constituted the core pathways involved in early neuronal development. These results suggest that the distinct association of risk genes with the neuronal lineage aligns with functional variations across neuronal maturation.

### Exploring neurological disorder-related gene expression in glial cell types

Glial cells play pivotal roles in maintaining nervous system homeostasis, providing support and protection to neurons, and participating in signal transmission. Dysfunction of glial cell types has been reported in several neurodegenerative disorders, such as Alzheimer’s disease and Parkinson’s disease. Nonetheless, the detailed trajectories of glial cell differentiation and their implications in neurological disorders remain unknown. As described previously, we mapped the risk genes for neurological disorders in a developmental trajectory of glial cell types (Fig. 2a). Astrocytes (C2, C27) exhibited significant enrichment of risk genes for developmental delay (C2, OR = 2.22, adjusted *p* value = 1.69 × 10^−4^) and autism (C27, OR = 1.96, adjusted *p* value = 3.67 × 10^−3^). The risk genes for Alzheimer’s disease were predominantly expressed in microglia (C13, OR = 3.83, adjusted *p* value = 3.96 × 10^−3^), emphasizing their potential role in the pathology or progression of Alzheimer’s disease. The risk genes for Parkinson’s disease were specifically enriched in fetal oligodendrocytes (C38) (OR = 3.22, adjusted *p* value = 2.53 × 10^−4^) and OPCs (C9, OR = 2.86, adjusted *p* value = 5.65 × 10^−6^; C32, OR = 1.89, adjusted *p* value = 2.92 × 10^−3^).

**Fig. 3:**
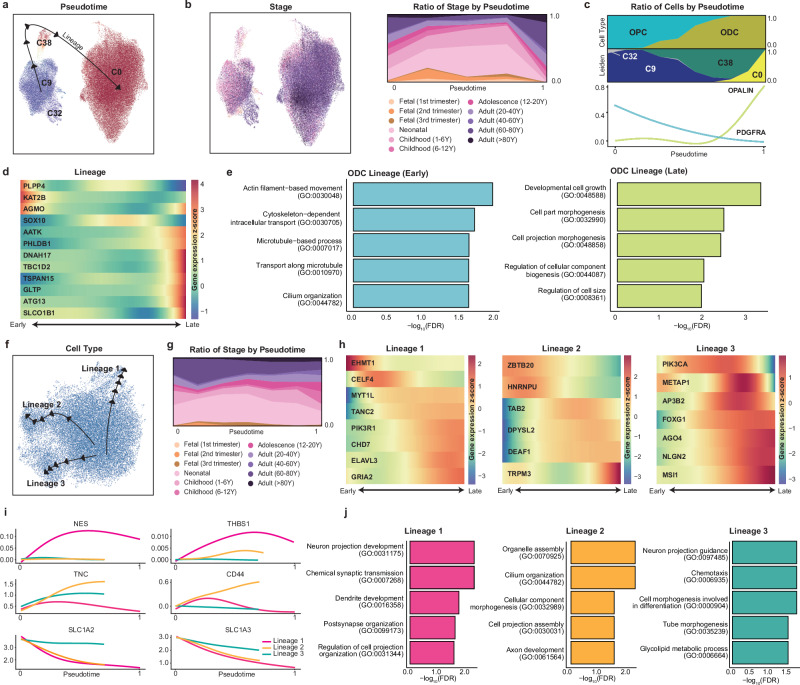
Cellular trajectories of neurological disorder risk genes in nonneuronal lineages. UMAP visualization of estimated developmental lineages in the oligodendrocyte group (C0, C8, C9, and C38), colored by pseudotime (**a**) and developmental stage (**b**). **c** Distribution of cells by major cell type and clustering across pseudotime and temporal patterns of PDGFRA and OPALIN. **d** Expression profiles of neurological disorder risk genes across pseudotime for different lineages. **e** Functional annotations for early- and late-lineage cells with significantly enriched biological processes with multiple comparisons by FDR. **f** UMAP visualization of the estimated developmental lineage in the astrocyte group (C2 and C29). **g** Distribution of cells by developmental stage across pseudotime. **h** Expression profiles of neurological disorder risk genes across pseudotime for each lineage. **i** Distinct expression patterns of known astrocyte marker genes in each lineage. **j** Functional annotations for each lineage with significantly enriched biological processes with multiple comparisons by FDR.

To further investigate the expression dynamics of these risk genes, we identified individual cellular trajectories for each cell type and their expression profiles. We found that oligodendrocyte progenitors diverged from radial glia and were distinct from neurons (Supplementary Fig. 4). Focusing on the dynamics of the oligodendrocyte lineage, we examined the differentiation of these cells starting from OPCs. Oligodendrocytes appeared to have a single trajectory from OPCs (C9 and C32), differentiating into fetal oligodendrocytes (C38) and mature oligodendrocytes (C0) (Fig. 3a). This trajectory was characterized by mixed developmental stages across the lineage pseudotime, suggesting the presence of both progenitor and mature cells throughout the postnatal period (Fig. 3b, c, Supplementary Fig. 5a–d). Risk genes specifically expressed in the oligodendrocyte lineage exhibited distinct peak expression patterns (Fig. 3d). In the earlier lineage, risk genes associated with abnormal brain morphology (AGMO) and anxiety disorders (KAT2B) were highly expressed. Earlier genes were involved in neuronal projections and intracellular cytoskeletal activity (Fig. 3e, Supplementary Table 4g). This included a microtubule-based process (GO:000701, FDR = 1.96 × 10^−2^) mediated by KAT2B, a risk gene for abnormal brain morphology. SOX10, a risk gene associated with developmental delay, was expressed at the highest level when OPCs differentiated into oligodendrocytes. Conversely, PHLDB1 spiked as oligodendrocytes matured, implying temporal variation in the expression of developmental delay risk genes during oligodendrocyte development. Two Parkinson’s disease risk genes presented distinct patterns, with PLPP4 expressed in initial OPC cells and DNAH17 expressed in late oligodendrocytes. Other genes expressed in the latter part of the lineage included anxiety disorder risk genes (AATK and TBC1D2), abnormal brain morphology genes (TSPAN15, GLTP, and SLCOB1), and an autism risk gene (ATG13). Cells at a later stage exhibited enrichment in pathways related to cell growth and morphogenesis (Fig. 3e, Supplementary Table 4h), suggesting a maturation process rather than rapid differentiation and transition of the cell state.

Astrocytes possess three distinct lineages, regardless of their developmental stage or cluster (Fig. 3f–j, Supplementary Fig. 5e–i, Supplementary Table 4i–k). These lineages were further characterized by the distinct expression of risk genes and known markers representing specific astrocyte subtypes^27–29^. In lineage 1, the autism risk gene EHMT1 was highly expressed at the earliest point, followed by the sequential peak of CELF4. The risk genes of developmental disorders (ELAVL3, GRIA2, and CHD7) presented high expression at the later stages of the lineage. This lineage was enriched in neuron projections and synaptic transmission pathways. Lineage 1 was enriched exclusively in the intermediate filament (IF) gene nestin (NES), which was also a marker of neural stem and progenitor cells. It was also distinctly expressed with THBS1, which is known to promote synaptogenesis^30^. Pathways enriched in this lineage are associated mainly with neuronal development, suggesting its neurodevelopmental role. In lineage 2, risk genes associated with developmental delay, such as ZBTB20 and HNRNPU, were highly expressed at the initial stage. In contrast, TAB2 expression peaked during the middle stage, whereas TRPM3 exhibited strong expression at the latter stage, suggesting distinct roles for each gene during cell maturation within the lineage. The autism risk genes involved in transcriptional regulation (DEAF1) and the cytoskeleton (DPYSL2) were strongly expressed at the end of the lineage. This lineage depicts the differentiation of fibrous astrocytes and is characterized by distinct expression of the fibrous astrocyte marker genes TNC and CD44, which are known to be involved in cell‒cell interactions and cell adhesion^31,32^. The cilium organization pathway (GO:0044782, FDR = 7.68 × 10^−2^) was also enriched in this lineage, where the cilium serves as a microtubule-based signaling device for various physiological functions of astrocytes. In lineage 3, PIK3CA, a risk gene for both autism and developmental delay, peaked in the middle part of the lineage. This was followed by the sequential expression of METAP1, a risk gene for autism and schizophrenia, along with AP3B2, a risk gene for developmental delay. The expression levels of other genes associated with developmental delay (FOXG2, NLGN2, and MSI1) peaked later in the lineage. Lineage 3 contains cells undergoing protoplasmic astrocyte differentiation. This lineage is characterized by constant expression of the glutamate transporter genes SLC1A2 and SLC1A3, which are marker genes of protoplasmic astrocytes^28,33^. These results suggest an association of risk genes with cellular maturation lineages in nonneuronal cells, indicating the temporal specificity of disorder risk.

### Multifaceted regulatory mechanisms governing early brain development

**Fig. 4:**
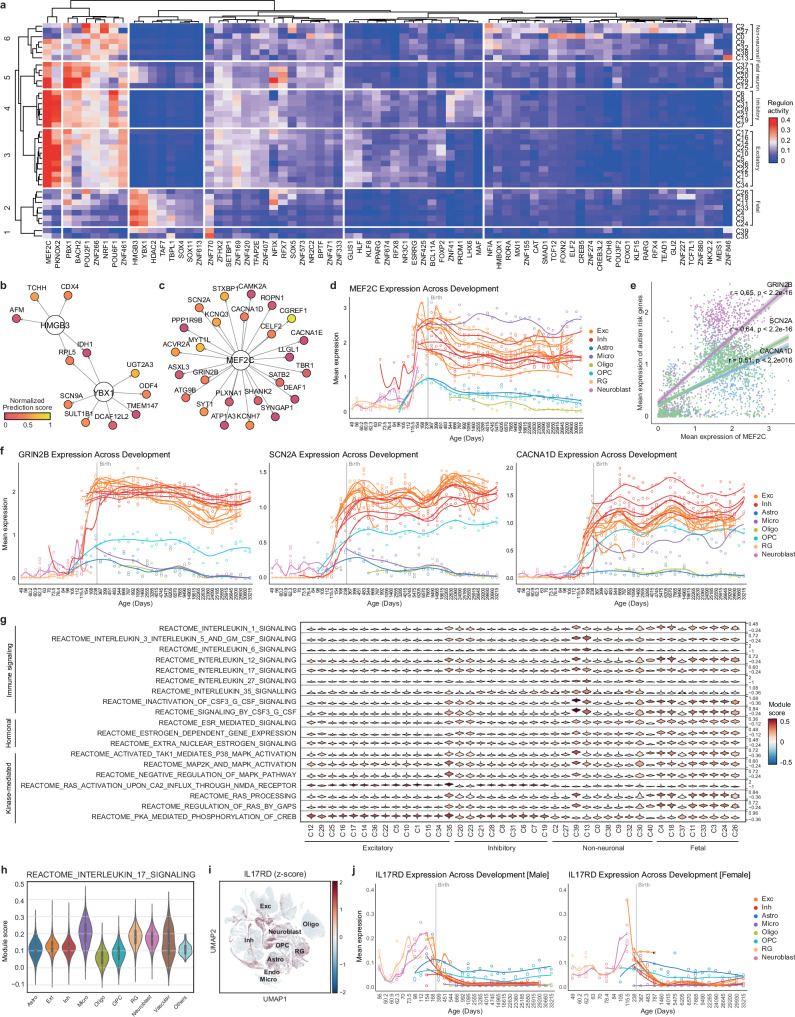
Regulatory landscape and pathway enrichment in early brain development. **a** Heatmap illustrating the regulon activities of transcription factors across clusters. Transcription factor-target networks depicting the regulation of glioblastoma risk genes by HMGB3 and YBX1 (**b**) and the regulation of autism risk genes by MEF2C (**c**). Prediction confidence was normalized from 0 to 1. The top 25 high-confidence targets for MEF2C are shown. **d** Expression of MEF2C over gestational days. The samplewise mean of log-normalized MEF2C expression was computed using a pseudobulk method. Clusters with at least 4,600 cells (C0–C22) were used. **e** Correlations between the samplewise means of log-normalized MEF2C expression and the expression of GRIN2B, SCN2A, and CACNA1D. **f** Expression of GRIN2B, SCN2A, and CACNA1D across developmental ages. **g** Violin plot displaying the pathway module scores as the average expression levels of pathway genes adjusted for control features. **h** Violin plot of pathway module scores across major cell types. **i** UMAP visualization of z score-normalized IL17RD expression. **j** Expression of IL17RD over gestational days, stratified by sex.

To deepen our understanding of the complex orchestration of brain development, we investigated the regulatory landscape of transcription factors, immune signaling, hormonal regulation, and kinase-mediated pathways. First, we predicted a cluster-specific gene regulatory network linking transcription factors to their putative target genes by integrating motif enrichment and gene coexpression analyses (Supplementary Table 5a). In total, 633 transcription factors were predicted to be activated in at least one cell type (Fig. 4a). Based on the predicted regulon activity of the transcription factors, the clusters were categorized into six different groups. From this, we identified canonical transcription factors for neurodevelopment, such as NFIA (astrocytes)^34,35^ and TCF12 (oligodendrocytes)^36^, confirming the validity of our findings.

Based on these results, we further elucidated the transcription factors implicated in neurological disorders. Fetal radial glia and neuroblast cells were enriched for HMGB3 and YBX1 (Group 2). These genes are involved in glioblastoma tumorigenesis and tumor growth^37,38^. We found that HMGB3 and YBX1 were predicted to target 11 risk genes for glioblastoma, including RPL5 and IDH1, as shared target genes (Fig. 4b). Fetal neurons appeared to have different transcription factors for later differentiation from radial glia and neuroblasts. Fetal neurons were regulated by MEF2C, PBX1, BACH2, POU2F1, NFIX, and RFX7 (Group 3). NFIX, known for its involvement in the differentiation of radial glia into the neuronal IPC^39^, and RFX7, known for its role in neural tube information^40^, exhibited distinct activities within this group. NFIX has been predicted to target 28 autism risk genes, including DIP2C and CSNK1E. MEF2C, which further displays potent activity in adult neurons, is a transcription factor that regulates multiple genes associated with autism^41^. We identified 47 autism-risk genes that were predicted to be targeted by MEF2C (Fig. 4c). The expression level of MEF2C increased in neurons across developmental stages, particularly in the fetal third trimester (Fig. 4d). This pattern was consistent with those of other autism risk genes, such as GRIN2B (correlation coefficient (r) = 0.65, *p* < 2.2e-16), SCN2A (*r* = 0.64, *p* < 2.2e-16), and CACNA1D (*r* = 0.51, *p* < 2.2e-16), suggesting a coordinated increase during the fetal third trimester (Fig. 4e, f). These results suggest that the activation of MEF2C plays a leading role in controlling the expression of autism-risk genes.

We further compared the signature genes of hormonal regulation and kinase-mediated and immune signaling pathways across cell types to elucidate their contributions to neurodevelopment (Fig. 4g, Supplementary Fig. 6, Supplementary Table 5b). Pathways associated with the sex hormone estrogen were more enriched in fetal neurons than in adult neurons, implying differential responsiveness to hormonal cues during neural maturation. Conversely, kinase activity, especially within the RAS pathways, was predominantly enriched in adult neurons compared with fetal neurons, suggesting its roles in neuronal survival and regeneration rather than in neurodevelopment.

Compared with other cell types, immune signaling pathways were highly enriched in fetal and adult microglia (Supplementary Table 5c), indicating their pivotal role in immune activities within the brain. In addition to microglia, radial glia, and neuroblasts were enriched in immune pathways, particularly interleukin (IL) signaling pathways (Fig. 4h). Previous studies have reported putative roles for IL-6 and IL-17A in the development of an autism-like behavioral phenotype in mouse models of maternal immune activation (MIA)^42,43^. Autism-like behavior in the MIA mouse model is mediated by IL-17 receptor expression in the brains of offspring^44^. While some of the IL-17 receptor genes (IL17RA, IL17RB, IL17RC, and IL17RE) were not distinctly expressed in the human fetal brain (Supplementary Fig. 7), IL17RD was elevated in fetal radial glia and neuroblasts (Fig. 4i), indicating its role in the prenatal risk of MIA. We further examined the onset of IL17RD expression and found sex-dependent differences (Fig. 4j, Supplementary Figs. 8, 9). In males, IL17RD expression in radial glia and neuroblasts began on approximately gestational Day 60, peaked between Days 112 and 154, and then decreased postnatally. In females, IL17RD expression began later, rising rapidly between gestational Days 84 and 105, peaking at approximately Day 115.5, and then decreasing after birth. As MIA offspring are known to exhibit male-biased behavioral abnormalities^45^, these findings may indicate that the differential regulation of IL17RD expression between males and females may contribute to the varying susceptibility to MIA.

### Identification of the cellular characteristics underlying glioblastoma

Glioblastoma is a lethal primary brain tumor characterized by intratumoral heterogeneity. A single-cell atlas may help in understanding the cellular heterogeneity underlying the clinical and molecular complexity of this disorder. We first examined whether the glioblastoma driver genes^46^ overlapped with cell type-specific genes. None of the cell types showed significant enrichment. These findings suggest that genomic associations might not fully characterize the molecular underpinnings of glioblastoma.

**Fig. 5:**
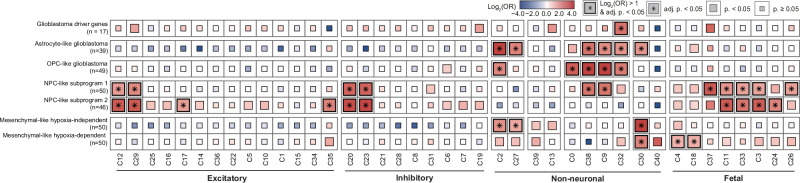
Cell type and temporal specificity in glioblastoma subtypes. Gene set enrichment test with driver genes and transcriptional signatures of glioblastoma. A one-sided Fisher’s exact test was used to compute statistics with multiple comparisons by Bonferroni correction.

Thus, we examined the cellular specificity of the signature gene sets previously defined to represent diverse cellular states in glioblastoma^47^ (Fig. 5, Supplementary Table 3c). Overall, the signature gene sets were aligned with the corresponding original cell types. In astrocyte-like glioblastoma, AQP4, a gene that regulates astrocytic process motility, and GFAP, the gene responsible for the cytoskeletal structure of astrocytes, mediated the enrichment of astrocyte clusters (C2, C27)^48,49^. For OPC-like glioblastoma, PLP1, PLLP, and BCAS1, which are known to be related to the ability of oligodendrocytes to form and maintain myelin in the central nervous system, mediated the enrichment of OPC lineages (C9, C32, and C38)^50,51^.

There was some overlap in the enrichment of the astrocyte cluster (C2) and OPC-like glioblastoma, as well as the OPC lineage clusters (C9, C32, C38) and astrocyte-like glioblastoma, demonstrating the shared nature among glial cell types^52^. Genes including BCAN, TTYH1, RAB31, GPM6B, PMP2, and PTPRZ1 were expressed in both lineages, contributing to this shared enrichment pattern. However, the degrees of enrichment were generally greater in astrocyte-like glioblastomas for astrocyte clusters and in OPC-like glioblastomas for OPC lineage clusters. For example, the astrocyte cluster (C2) was more enriched in astrocyte-like glioblastoma (OR = 23.40, adjusted *p* value = 2.0 × 10^−17^) than in OPC-like glioblastoma (OR = 5.63, adjusted *p* value: 2.0 × 10^−4^). Similarly, the OPC lineage cluster (C38) was more strongly enriched in OPC-like glioblastoma (OR = 54.46, adjusted *p* value = 5.1 × 10^−32^) than in astrocyte-like glioblastoma (OR = 7.84, adjusted *p* value = 8.1 × 10^−3^).

Neural progenitor cell (NPC)-like glioblastoma subprograms were significantly enriched in fetal neurons and fetal cell types. NPC-like subprogram 1 was enriched for OPCs and oligodendrocytes. Among the signature genes, BCAN, DLL3, OLIG1, LRRN1, TCF12, and TNR were expressed specifically in these cell types. In contrast, NPC-like subprogram 2 demonstrated significant overlap for layer 4–6 excitatory neurons (C17) and layer-unspecific excitatory neurons (C35). TCF4 was included among the signature genes of NPC-like subprogram 2, emphasizing its regulatory function in tumor progression.

Mesenchymal-like glioblastoma exhibited different enrichment patterns between hypoxia-independent and hypoxia-dependent subgroups. While both were enriched in vascular cells (C30), the hypoxia-independent subgroup was enriched in astrocytes (C2, C27), and the hypoxia-dependent subgroup was enriched in radial glial subtypes expressing HES1 (C4, C18), implying a possible resemblance of each astrocyte and radial glial subtype to mesenchymal-like glioblastoma. These results highlight the importance of investigating cell type-specific perspectives and studying brain cell types across primary developmental stages to gain better clinical insights into disease initiation and potential treatment options.

## Discussion

In this study, we constructed a comprehensive single-cell atlas of the developing human brain to investigate the cellular and temporal specificity of genes implicated in neurological disorders. Atlas-level integration enables detailed functional and molecular profiling of various cell types across multiple human samples. By analyzing 393,060 single brain cells, our research revealed the complex cellular compositions and dynamic changes during early brain development.

Neurological disorders are characterized by high levels of genetic heterogeneity, and multiple genes may be associated with a single disorder^53,54^. Despite the enormous success of large-scale genomic studies, such as GWASs, these associations require appropriate interpretation in a biological and genomic context. Consequently, deciphering the functional convergence of risk genes is essential for comprehending disease pathophysiology and identifying potential therapeutic targets^55^. Our single-cell atlas can be useful for thorough analyses of risk genes associated with neurological disorders that may occur during brain development. We detected distinct expression patterns of autism risk genes, including FOXP2, highlighting the temporal regulation of excitatory and inhibitory neuronal lineages. The PD risk genes PLPP4 and DNAH17 were shown to be expressed at distinct time points during oligodendrocyte differentiation, implying that temporal specificity also appears in glial cells. Moreover, the atlas enabled the exploration of novel disease mechanisms. For example, MEF2C, a transcription factor activated during the transition from prenatal to postnatal neurons, aligns expression patterns with autism risk genes. In addition, the distinct sex difference in the onset of IL17RD expression may indicate that it is a putative target contributing to varying susceptibility to MIA. Furthermore, certain cellular states in glioblastoma closely resemble fetal-stage cell types despite their origin in adults, implying the necessity of investigating the characteristics leading to this similarity.

Extensive research has focused on the cell type-specific nature of neuronal diseases, with a primary focus on stage-specific neuronal cells. However, to further enhance clinical investigations and therapeutics, it is crucial to consider the temporal and cellular specificity of neuronal disorders, as well as nonneuronal cell types, which also exhibit temporal specificity across cellular maturation. Moreover, our study of neuronal disorders revealed the significant enrichment of fetal cell types. These findings suggest that the determination of disorders may be strongly influenced during the prenatal stage. Therefore, we propose that studying temporal specificity across brain development warrants a more robust investigation.

Although our single-cell atlas leverages a large number of postmortem samples to explore the developing human brain, our study has several limitations. Despite the use of data from various donors, our atlas may not encompass the full spectrum of individual variability in developmental trajectories. For example, the neonatal and early childhood periods are crucial for assessing synaptic pruning and the maturation of neural circuits; nevertheless, the availability of human postmortem brain samples from these stages is limited. A more extensive and diverse collection of samples would enhance our understanding of the pivotal role of genetic constitution in early brain development and its impact on gene expression^5^. Additionally, owing to the predominant focus on cortical regions in postnatal datasets, there are limitations in representing diverse brain regions across all developmental stages. Addressing these gaps will require concerted efforts to collect and analyze samples from these critical developmental stages and diverse brain regions to enrich our understanding of brain development and its implications for neurological disorders. Nevertheless, we anticipate that this atlas would provide a useful foundation for addressing the complexity of brain development and neurological disorders.

## Supplementary information

Supplementary Information

Supplementary Table 1

Supplementary Table 2

Supplementary Table 3

Supplementary Table 4

Supplementary Table 5

## Data Availability

The processed data and the CellTypist model for label transfer are publicly available at Zenodo (10.5281/zenodo.10939707). Plots illustrating the expression profiles of 3380 neurological disorder risk genes across the atlas are also provided. Further availability of the data can be requested from the corresponding author.
