# Supplementary material for: An integrative single-cell atlas for exploring the cellular and temporal specificity of genes related to neurological disorders during human brain development
Source: Exp Mol Med. 2024 Oct 3;56(10):2271–82. doi: 10.1038/s12276-024-01328-6 (PMC11541755; doi:10.1038/s12276-024-01328-6)
Supplement: Supplementary file 1 — Supplementary Information [file 12276_2024_1328_MOESM1_ESM.pdf]

Supplementary Information

This file includes:

Supplementary Methods

Supplementary Fig. 1 – 9

Supplementary Table 1 – 5

Supplementary Methods

Evaluation of batch correction results

During the integration process, we used the `scvi.setup_anndata()` function. We added 'Dataset,' 'Assay,' and 'Library' as the `categorical_covariate_keys,` removing the potential cause of the technical batch effect by the difference of the dataset, assay (10x chromium v2 or 10x chromium v3), and library (scRNA or snRNA). To validate the batch correction results, we calculated and compared the average silhouette width (ASW) for three atlas anndata objects: an unintegrated, highly variable gene (HVG) filtered with batch-aware feature selection and our batch-corrected dataset using scVI. We applied the `silhouette_batch()` function from the scib[1] package for each categorical covariate key, with them assigned as the 'batch_key'.

Gene set enrichment test for cell type validation

A gene set enrichment test was performed for the established cell type marker genes from the previous single-cell transcriptomic study of the developing brain[2]. The background genes for the enrichment test were set to include all the genes included in the single-cell dataset of the previous study. Marker genes for each annotated cluster EN (excitatory neurons from postnatal samples), EN-fetal-early (excitatory neurons from early fetal samples), EN-fetal-late (excitatory neurons for late fetal samples), CGE-derived inhibitory neurons (IN-CGE), MGE-derived inhibitory neurons (IN-MGE), astrocytes, microglia, oligodendrocytes, OPC, pericytes, VSMC, endothelial cells, RG, and IPC were collected from the supplementary data provided. This enrichment test included cluster-specific differentially expressed genes (DEGs) with a threshold of FDR <0.05, log2 fold change >0.2, and >25% of cells within the cluster expressing the gene. A one-sided Fisher's exact test with multiple comparisons was applied.

Collation of neurological disorders and glioblastoma risk genes

Risk genes of neurodevelopmental disorders, including autism, epilepsy, or developmental delay, were selected. For autism, we utilized risk genes identified in large-scale exome studies. 185 genes with the enrichment of protein-truncating

variants (PTVs), missense variants, and copy number variants (CNVs) in 20,627 autism cases qualified multiple comparisons at FDR <0.05 were selected from Fu et al.[3]. In addition, we utilized 373 risk genes from the meta-analysis of cohorts ascertained for developmental delay (DD). We also chose 102 genes that are enriched for 11,986 autism cases at FDR <0.1 from Satterstrom et al.[4]. Epilepsy risk genes were sourced from the Epi25 dataset, comprising a compilation of 20,979 cases and 33,444 controls from 59 global research cohorts. A total of 140 genes were selected from the summary statistics (https://epi25.broadinstitute.org/results) based on the enrichment of PTVs or damaging missense variants in case subjects (p-value <0.01).

For neuropsychiatric disorders, we chose the risk genes for anxiety disorder, bipolar disorder, major depression, and schizophrenia. We selected 692 genes from the anxiety-associated genomic loci in the GWAS catalog (EFO ID: EFO_0006788). We selected 58 bipolar disorder risk genes from the BipEx dataset, encompassing data from 14,210 cases and 14,422 controls. Genes significantly enriched for PTVs or damaging missense variants (p-value <0.01) in cases were subset from the summary statistics (https://bipex.broadinstitute.org). We obtained 450 major depression-associated genes from a GWAS analysis of 88,316 cases and 902,757 controls[5]. Schizophrenia-related genes were sourced from exome and GWAS analysis. We utilized the schizophrenia exome meta-analysis consortium (SCHEMA) dataset, which includes data from 24,248 cases, 97,322 controls, and 3,402 parent-proband trios. Gene selection (n = 32) was guided by FDR <0.05. Additionally, 287 schizophrenia-associated loci and 2,132 genes were obtained from Trubetskoy et al.[6], involving 76,755 individuals with schizophrenia and 243,649 controls.

For neurodegenerative disorders, we focused on genes associated with Alzheimer’s and Parkinson’s diseases. We selected 76 genes associated with Alzheimer's disease from a recent GWAS comprising 111,326 cases and 677,663 controls[7]. Parkinson’s disease genes (n=423) were retrieved based on GWAS loci reported in the GWAS catalog (EFO ID: MONDO_0005180).

Furthermore, genes associated with neurological conditions in response to trauma exposure (EFO ID: EFO_0008483), vascular brain injury (EFO ID: EFO_0006791), and abnormal brain morphology (EFO ID: HP_0012443) were included. These were identified from GWAS loci reported in the GWAS catalog, with a

specific number of undisclosed genes. For glioblastoma, 17 glioblastoma driver genes were selected[8]. Gene set signatures for specific cellular states in glioblastoma (Astrocyte-like, OPC-like, NPC-like subprogram 1, NPC-like subprogram 2, Mesenchymal-like hypoxia-independent, and Mesenchymal-like hypoxia-dependent) were obtained from the meta-module gene list, identified to be recurrent across tumors indicating global characterization of intra-tumoral heterogeneity[9].

Pseudo-time and trajectory analysis

Pseudo-time analysis was performed using Palantir[10]. Subset of each cell type of interest was reprocessed before the analysis. Samples with <100 cells were excluded from the pre-processing and integration steps to ensure robustness. To mitigate the batch effect, 5,000 highly variable genes were selected from each sample and integrated using scvi-tools[11].

Diffusion maps were derived from batch-corrected embeddings, and the resulting components were projected onto Uniform Manifold Approximation and Projection (UMAP). Pseudo-time computation and trajectory construction were conducted by designating cells with the minimum age of the premature cell type as the initial cell for each group. For instance, in the neuronal group, one of the earliest radial glial cells was chosen as the initial state. Similarly, in the oligodendrocyte and astrocyte groups, one of the OPC cells and astrocytes with the minimum age was considered the initial states. The endpoints of the trajectories were determined automatically. Differentiation potential was estimated by quantifying the entropy and pseudo-time distance of each cell from the initial state. Gene trends were subsequently computed for each lineage, and gene expression was illustrated across pseudo-time for temporal investigation.

Inference of gene regulatory networks and enriched signaling pathways

To facilitate gene regulatory network inference, transcriptionally similar cells were categorized into meta-cells using SEACells[12] (v0.3.3). Following the designation of one meta-cell for every 75 single cells, 393,060 cells were aggregated into 5,000 meta-cells. In the initialization step, the embedding matrix generated by scVI was used to

compute the kernel for the meta-cells and prioritize the top 10 eigenvalues. Subsequently, we constructed the kernel matrix and conducted an archetypal analysis. The minimum and maximum iterations were set to 10 and 100 in the model fitting step, with a convergence threshold of 0.01125. Within each SEACell, cellular aggregation was achieved by summing the log-normalized expression of all constituent cells, generating aggregated counts.

Transcription factor regulatory networks were inferred using pySCENIC[13] (v0.12.1). Log-transformed counts from the SEACells were used as an input matrix, considering only protein-coding genes. Adjacencies between the transcription factors and their targets were inferred using the GRNBoost2 algorithm. Regulon prediction for 1,892 transcription factors was performed based on the motifs of the transcription factors and putative promoter regions of the target genes obtained from the cisTarget database (version 9), covering 10kb around the transcription start site, 500 bases upstream, and 100 bases downstream. The correlation between transcription factor and target genes was calculated using the entire set of cells, including those with zero expression. Cellular enrichments of regulons per SEACell were determined with an AUC threshold of 0.05, and the resulting regulon activities calculated for each SEACell were matched to the Leiden clusters possessing the highest cell counts. The regulon activities for transcription factors with a minimum mean of 0.02 and a variance of 0.001 across Leiden clusters were visualized as a heatmap using the R package ComplexHeatmap (v2.15.1). Gene sets representing hormonal regulation, kinase-mediated, and immune signaling pathways were obtained from the Reactome database[14]. Module scores were computed by averaging the expression levels of genes within each gene set and subtracting the average expression of a reference set of genes.

Building an annotation prediction model with CellTypist

We built a prediction model using the CellTypist package (v1.2.0)[15] with a two-pass data training approach for further annotation. In the first pass, we implemented stochastic gradient descent (SGD) logistic regression with mini-batch training to identify features for model building. We ranked the top 100 genes associated with each Leiden cluster by their absolute regression coefficients and selected these as features.

Using the filtered data with the selected features, we performed logistic regression in the second pass with default parameters.

References

1 Luecken, M. D. *et al.* Benchmarking atlas-level data integration in single-cell genomics. *Nature Methods* **19**, 41-50 (2022). https://doi.org/10.1038/s41592-021-01336-8

2 Zhu, K. *et al.* Multi-omic profiling of the developing human cerebral cortex at the single-cell level. *Science Advances* **9**, eadg3754 (2023). https://doi.org/doi:10.1126/sciadv.adg3754

3 Fu, J. M. *et al.* Rare coding variation provides insight into the genetic architecture and phenotypic context of autism. *Nat Genet* **54**, 1320-1331 (2022). https://doi.org/10.1038/s41588-022-01104-0

4 Satterstrom, F. K. *et al.* Large-Scale Exome Sequencing Study Implicates Both Developmental and Functional Changes in the Neurobiology of Autism. *Cell* **180**, 568-584 e523 (2020). https://doi.org/10.1016/j.cell.2019.12.036

5 Meng, X. *et al.* Multi-ancestry genome-wide association study of major depression aids locus discovery, fine mapping, gene prioritization and causal inference. *Nat Genet* **56**, 222-233 (2024). https://doi.org/10.1038/s41588-023-01596-4

6 Trubetskoy, V. *et al.* Mapping genomic loci implicates genes and synaptic biology in schizophrenia. *Nature* **604**, 502-508 (2022). https://doi.org/10.1038/s41586-022-04434-5

7 Bellenguez, C. *et al.* New insights into the genetic etiology of Alzheimer's disease and related dementias. *Nat Genet* **54**, 412-436 (2022). https://doi.org/10.1038/s41588-022-01024-z

8 Bailey, M. H. *et al.* Comprehensive Characterization of Cancer Driver Genes and Mutations. *Cell* **173**, 371-385.e318 (2018). https://doi.org/10.1016/j.cell.2018.02.060

9 Neftel, C. *et al.* An Integrative Model of Cellular States, Plasticity, and Genetics for Glioblastoma. *Cell* **178**, 835-849.e821 (2019). https://doi.org/10.1016/j.cell.2019.06.024

10 Wei, X. *et al.* Integrative analysis of single-cell embryo data reveals transcriptome signatures for the human pre-implantation inner cell mass. *Dev Biol* **502**, 39-49 (2023). https://doi.org/10.1016/j.ydbio.2023.07.004

11 Lopez, R., Regier, J., Cole, M. B., Jordan, M. I. & Yosef, N. Deep generative modeling for single-cell transcriptomics. *Nat Methods* **15**, 1053-1058 (2018). https://doi.org/10.1038/s41592-018-0229-2

12 Persad, S. *et al.* SEACells infers transcriptional and epigenomic cellular states from single-cell genomics data. *Nat Biotechnol* **41**, 1746-1757 (2023). https://doi.org/10.1038/s41587-023-01716-9

13 Van de Sande, B. *et al.* A scalable SCENIC workflow for single-cell gene regulatory network analysis. *Nat Protoc* **15**, 2247-2276 (2020). https://doi.org/10.1038/s41596-020-0336-2

14 Jassal, B. *et al.* The reactome pathway knowledgebase. *Nucleic Acids Res* **48**, D498-d503 (2020). https://doi.org/10.1093/nar/gkz1031

15 Domínguez Conde, C. *et al.* Cross-tissue immune cell analysis reveals tissue-specific features in humans. *Science* **376**, eabl5197 (2022). https://doi.org/10.1126/science.abl5197

Supplementary Figures

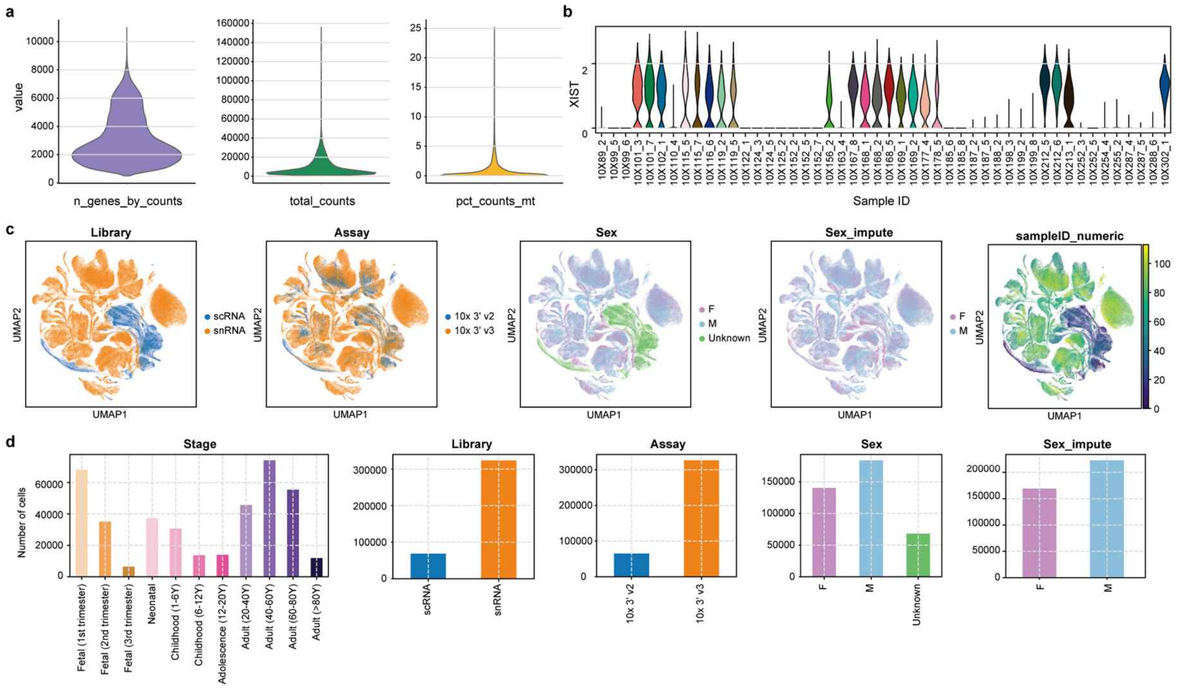

Supplementary Fig. 1. Comprehensive overview of quality metrics and sample information in single-cell atlas. a. Violin plot illustrating quality metrics, including the number of genes with at least one count per cell (n_genes_by_counts), the total number of counts per cell (total_counts), and the percentage of counts in mitochondrial genes (pct_counts_mt). **b.** Violin plot for log-normalized XIST expression for samples lacking sex information. **c.** UMAP of the atlas, colored by library, assay, sex, imputed sex (sex_impute), and a numeric representation of sample IDs (sampleID_numeric). **d.** The number of cells categorized by developmental stage, library, assay, sex, and imputed sex.

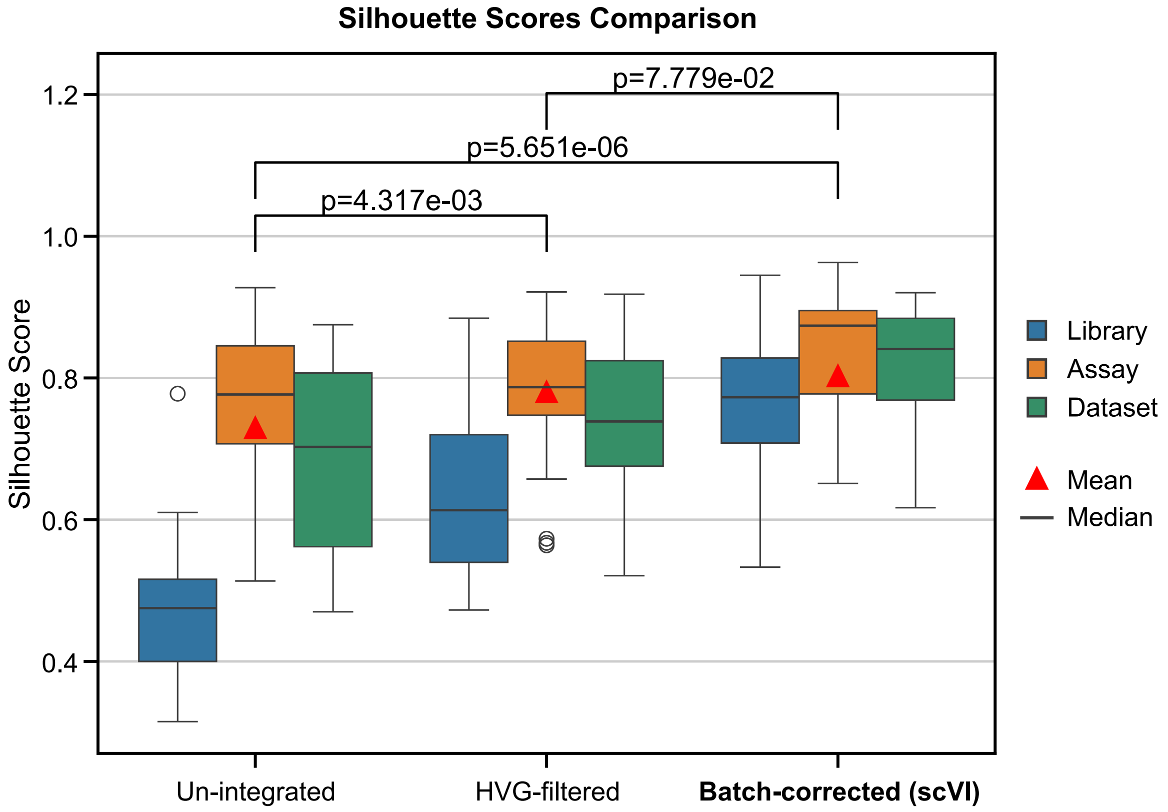

Supplementary Fig. 2. Comparison of silhouette scores for each technical batch key. Boxplot of absolute silhouette width (ASW) of clusters of un-integrated datasets, highly variable gene (HVG) filtered dataset, and the batch-corrected dataset with scVI tools in the aspect of the datasets, assays (10x chromium v2 and 10x chromium v3), and libraries (scRNA and snRNA). An absolute ASW close to 0 indicates poor batch mixing, while a value close to 1 indicates optimal mixing.

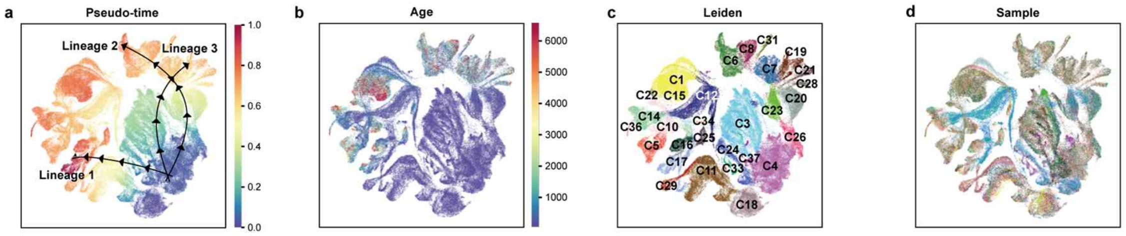

Supplementary Fig. 3. Estimated developmental lineages in neuronal cell types. a-d. UMAP visualizations of neuronal cell types colored by (a) estimated pseudo-time, (b) gestational age, (c) cluster, and (d) sample ID.

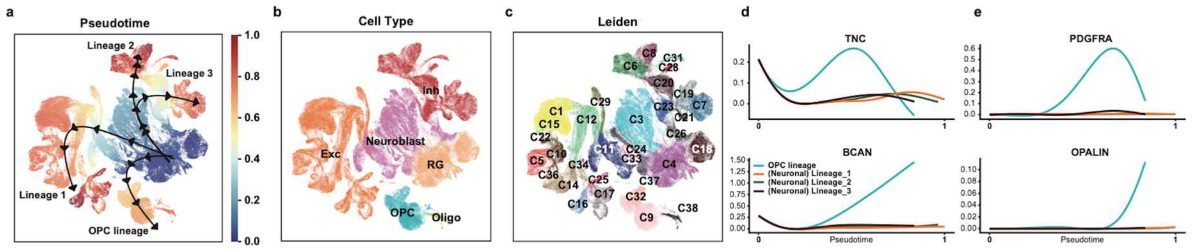

Supplementary Fig. 4. Estimated developmental lineages of neurons and OPC. a-c. UMAP visualizations of cells colored by (a) estimated pseudo-time, (b) cell type, and (c) cluster. **d.** Temporal expression patterns of OPC precursors marker genes. **e.** Temporal expression of OPC and oligodendrocyte marker genes.

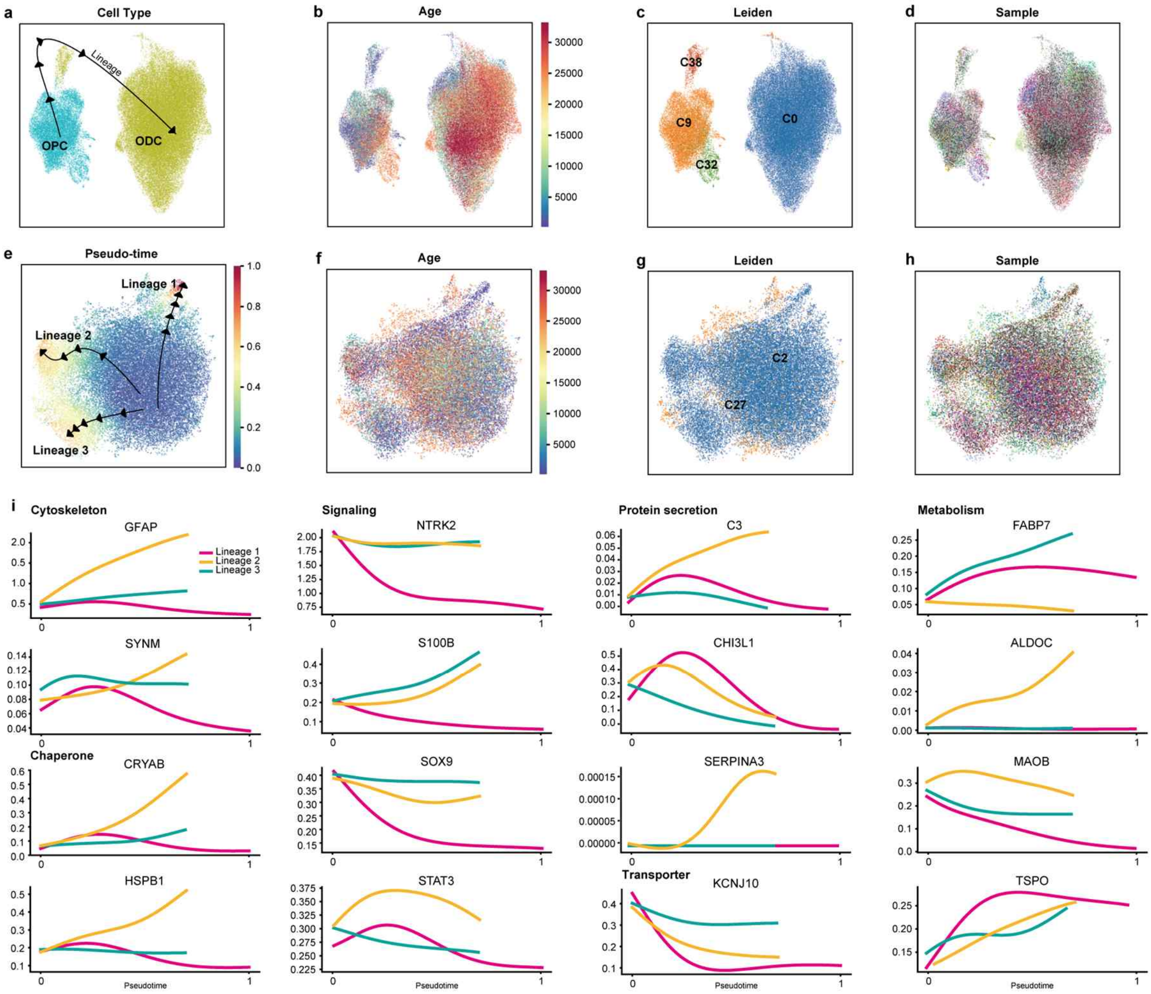

Supplementary Fig. 5. Estimated developmental lineages in non-neuronal cell types. a-d. UMAP visualizations of oligodendrocyte-lineage cell types colored by (a) cell type, (b) gestational age, (c) cluster, and (d) sample ID. **e-h.** UMAP visualizations of astrocytes colored by (e) estimated pseudo-time, (f) gestational age, (g) cluster, and (h) sample ID. **i.** Temporal expression patterns of genes associated with function enriched in reactive astrocytes. Expression of genes related to cytoskeleton organization (Cytoskeleton), chaperone activity (Chaperone), cell signaling (Signaling), secretion of proteins (Protein secretion), ion transporters (Transporter), and metabolism (Metabolism) of each lineage is depicted.

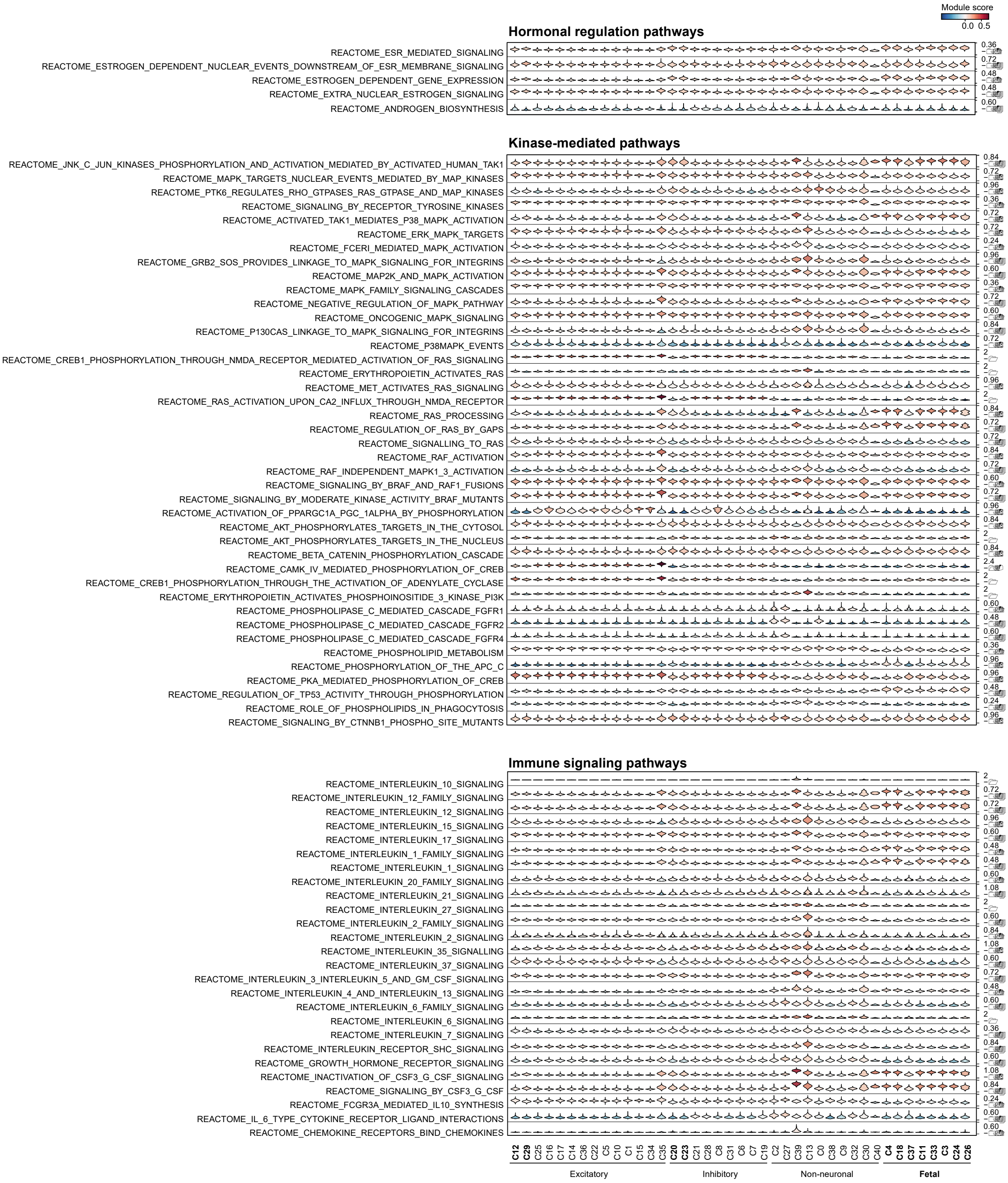

Supplementary Fig. 6. Pathway enrichment in early brain development. Violin plot displaying pathway module scores as the average expression level of pathway genes adjusted for control features.

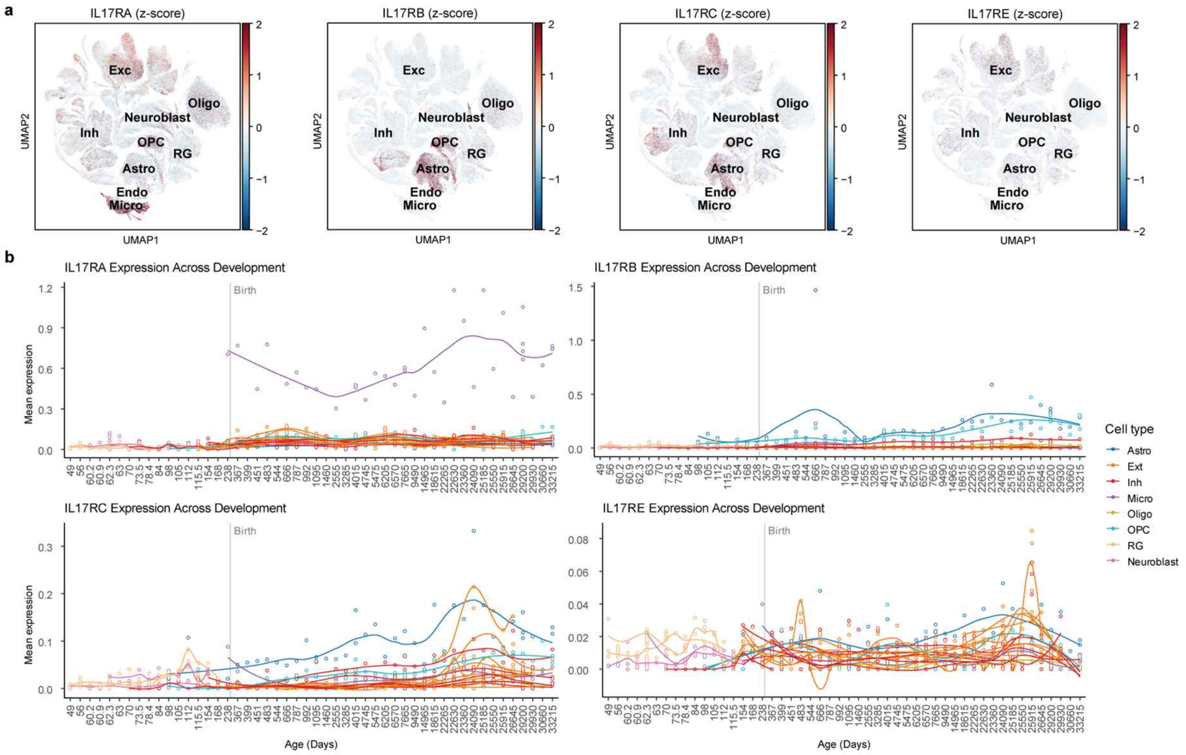

Supplementary Fig. 7. Expression profiles of IL-17 receptor genes (IL17RA, IL17RB, IL17RC, IL17RE). a. UMAP visualization of z-score normalized IL-17 receptor gene expression. **b.** Expression of IL-17 receptor genes over gestational days. The sample-wise mean of log-normalized gene expression was computed using a pseudo-bulk method. Clusters with at least 4,600 cells (C0-C22) were used.

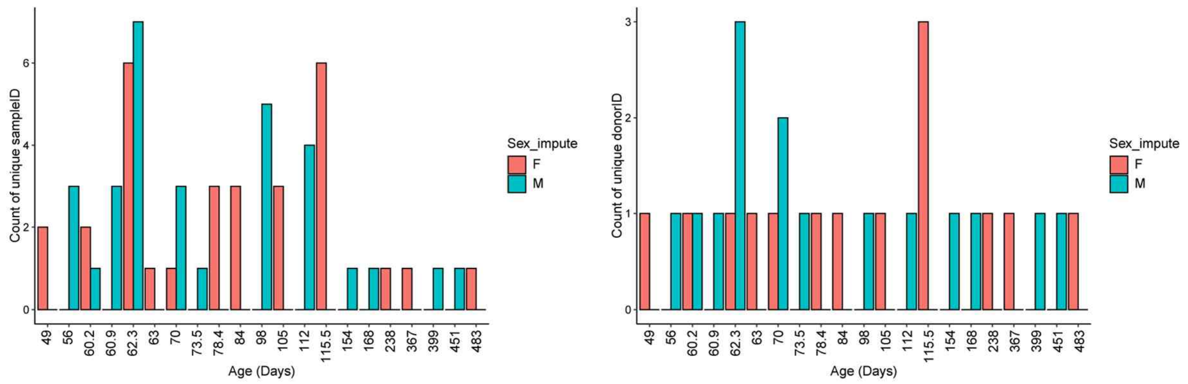

Supplementary Fig. 8. Distribution of samples and donors by sex across development. The bars represent the number of samples and the number of donors collected from male and female donors at different gestational periods. Samples at prenatal stages and the prenatal-to-postanal transition phase are included in the plot.

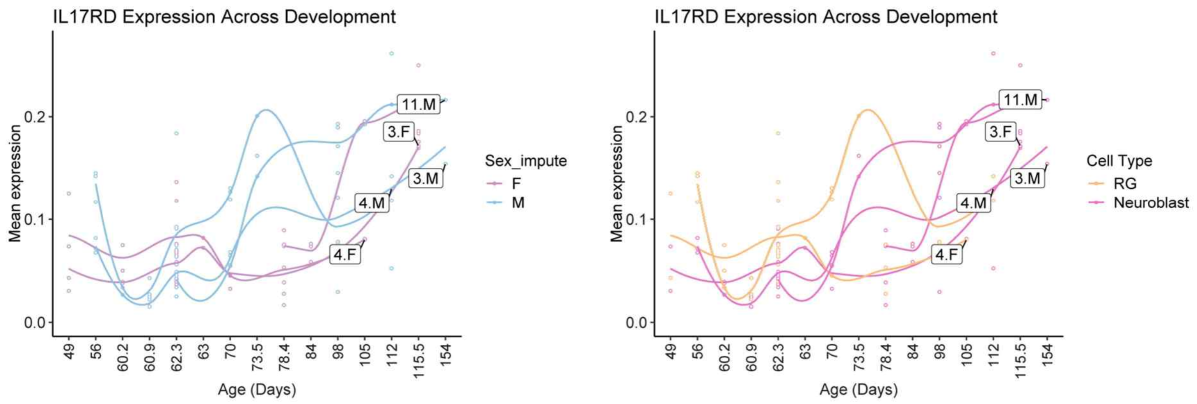

Supplementary Fig. 9. IL17RD expression in radial glia and neuroblasts by sex across development. Expression of IL17RD over gestational days, colored by imputed sex and cell types. The sample-wise mean of log-normalized gene expression was computed using a pseudo-bulk method. Clusters for radial glia and neuroblasts with at least 4,600 cells (C3, C4, C11) were used.

Supplementary Tables

Supplementary Table 1. Data information

Supplementary Table 1a. Dataset information

Supplementary Table 1b. Sample information

Supplementary Table 2. Identification of cluster-specific DEGs and validation

Supplementary Table 2a. Wilcoxon rank sum test result for cluster-specific DEGs

Supplementary Table 2b. Fisher's exact test with previously identified cell type markers

Supplementary Table 3. Association of clusters with neurological disorders

Supplementary Table 3a. Neurological disorder genes with details on DEG and lineage associations

Supplementary Table 3b. Fisher's exact test with neurological disorder risk genes

Supplementary Table 3c. Fisher's exact test with cellular states of glioblastoma

Supplementary Table 4. Pathway enrichment analysis for trajectory lineages

Supplementary Table 4a. Gene Ontology terms enriched for neuronal lineage 1

Supplementary Table 4b. Gene Ontology terms enriched for neuronal lineage 2

Supplementary Table 4c. Gene Ontology terms enriched for neuronal lineage 3

Supplementary Table 4d. Gene Ontology terms enriched for early cells of lineage 1

Supplementary Table 4e. Gene Ontology terms enriched for middle cells of lineage 1

Supplementary Table 4f. Gene Ontology terms enriched for late cells of lineage 1

Supplementary Table 4g. Gene Ontology terms enriched for early cells of oligodendrocyte lineage

Supplementary Table 4h. Gene Ontology terms enriched for late cells of oligodendrocyte lineage

Supplementary Table 4i. Gene Ontology terms enriched for astrocyte lineage 1

Supplementary Table 4j. Gene Ontology terms enriched for astrocyte lineage 2

Supplementary Table 4k. Gene Ontology terms enriched for astrocyte lineage 3

Supplementary Table 5. Multifaceted analysis of regulatory mechanisms

Supplementary Table 5a. Predicted activated transcription factor list per cluster

Supplementary Table 5b. Signatures of immune signaling, hormonal regulation, and kinase-mediated pathways

Supplementary Table 5c. Wilcoxon rank sum test for cell-type-wise comparison of pathway scores
